# Phase I Clinical Evaluation of Designed Ankyrin Repeat Protein [^99m^Tc]Tc(CO)_3_-(HE)_3_-Ec1 for Visualization of EpCAM-Expressing Lung Cancer

**DOI:** 10.3390/cancers16162815

**Published:** 2024-08-10

**Authors:** Roman Zelchan, Vladimir Chernov, Anna Medvedeva, Anastasia Rybina, Olga Bragina, Elizaveta Mishina, Mariia Larkina, Ruslan Varvashenya, Anastasia Fominykh, Alexey Schulga, Elena Konovalova, Anzhelika Vorobyeva, Anna Orlova, Liubov Tashireva, Sergey M. Deyev, Vladimir Tolmachev

**Affiliations:** 1 Department of Nuclear Medicine, Cancer Research Institute, Tomsk National Research Medical Center, Russian Academy of Sciences, 634055 Tomsk, Russia; chernov1962@gmail.com (V.C.); medvedeva@tnimc.ru (A.M.); pankovaan@mail.ru (A.R.); rungis@mail.ru (O.B.); liza.mishina.00@inbox.ru (E.M.); 2 Research Centrum for Oncotheranostics, Research School of Chemistry and Applied Biomedical Sciences, Tomsk Polytechnic University, 634050 Tomsk, Russia; mr.varvashenya@mail.ru (R.V.); anastasia.527@yandex.ru (A.F.); schulga@gmail.com (A.S.); elena.ko.mail@gmail.com (E.K.); biomem@mail.ru (S.M.D.); 3 Department of Pharmaceutical Analysis, Siberian State Medical University, 634050 Tomsk, Russia; 4 Shemyakin-Ovchinnikov Institute of Bioorganic Chemistry of the Russian Academy of Sciences, 117997 Moscow, Russia; 5 Department of Immunology, Genetics and Pathology, Uppsala University, 75185 Uppsala, Sweden; anzhelika.vorobyeva@igp.uu.se (A.V.); vladimir.tolmachev@igp.uu.se (V.T.); 6 Department of Medicinal Chemistry, Uppsala University, 75185 Uppsala, Sweden; anna.orlova@ilk.uu.se; 7 Laboratory of Molecular Therapy of Cancer, Cancer Research Institute, Tomsk National Research Medical Center, Russian Academy of Sciences, 634055 Tomsk, Russia; lkleptsova@mail.ru

**Keywords:** [^99m^Tc]Tc(CO)_3_-(HE)_3_-Ec1, single-photon emission computed tomography, lung cancer

## Abstract

**Simple Summary:**

The presented article is devoted to the study of the safety, biodistribution and absorbed doses of a new radiopharmaceutical for diagnostics and evaluation of the treatment efficiency of tumors with hyperexpression of epithelial cell adhesion molecules by single-photon emission computed tomography. The results of the study showed the safety and tolerability of [^99m^Tc]Tc(CO)_3_-(HE)_3_-Ec1 in patients with lung tumors. The absorbed dose values for [^99m^Tc]Tc(CO)_3_-(HE)_3_-Ec1 SPECT are also within the acceptable limits. Also, [^99m^Tc]Tc(CO)_3_-(HE)_3_-Ec1 SPECT showed primary diagnostic efficiency in visualizing tumors with EpCAM expression and regional metastases. The promising use of [^99m^Tc]Tc(CO)_3_-(HE)_3_-Ec1 will allow the selection of patients for targeted immunotherapy of tumors and evaluation of the efficiency of such treatment.

**Abstract:**

A high level of EpCAM overexpression in lung cancer makes this protein a promising target for targeted therapy. Radionuclide visualization of EpCAM expression would facilitate the selection of patients potentially benefiting from such treatment. Single-photon computed tomography (SPECT) using ^99m^Tc-labeled engineered scaffold protein DARPin Ec1 has shown its effectiveness in imaging tumors with overexpression of EpCAM in preclinical studies, providing high contrast just a few hours after injection. This first-in-human study aimed to evaluate the safety and distribution of [^99m^Tc]Tc(CO)_3_-(HE)_3_-Ec1 in patients with primary lung cancer. Twelve lung cancer patients were injected with 300.7 ± 103.2 MBq of [^99m^Tc]Tc(CO)_3_-(HE)_3_-Ec1. Whole-body planar imaging (at 2, 4, 6 and 24 h after injection) and SPECT/CT of the lung (at 2, 4, and 6 h) were performed. The patients’ vital signs and possible side effects were monitored up to 7 days after injection. The patients tolerated the injection of [^99m^Tc]Tc(CO)_3_-(HE)_3_-Ec1 well, and their somatic condition remained normal during the entire follow-up period. There were no abnormalities in blood and urine tests after injection of [^99m^Tc]Tc(CO)_3_-(HE)_3_-Ec1. The highest absorbed doses were in the kidneys, liver, pancreas, thyroid, gallbladder wall, and adrenals. There was also a relatively high accumulation of [^99m^Tc]Tc(CO)_3_-(HE)_3_-Ec1 in the small and large intestines, pancreas and thyroid. According to the SPECT/CT, accumulation of [^99m^Tc]Tc(CO)_3_-(HE)_3_-Ec1 in the lung tumor was found in all patients included in the study. Intensive accumulation of [^99m^Tc]Tc(CO)_3_-(HE)_3_-Ec1 was also noted in regional metastases. [^99m^Tc]Tc(CO)_3_-(HE)_3_-Ec1 can potentially be considered a diagnostic tracer for imaging EpCAM expression in lung cancer patients and other tumors with overexpression of EpCAM.

## 1. Introduction

Lung cancer is still one of the most common cancers worldwide. The incidence of lung cancer has increased over the past decade, among both men and women [1]. In 2018, the Global Cancer Observatory (GLOBOCAN) estimated that 2.09 million new cases (11.6% of total cancer cases) and 1.76 million deaths (18.4% of total cancer deaths) were reported [2]. It should be noted that smoking leads to 85–90% of all lung cancer cases [3]. In general, lung cancer is divided into two main types: non-small cell lung cancer (NSCLC, 85% of patients) and small cell lung cancer (15% of patients). According to the WHO classification, there are three main types of NSCLC: adenocarcinoma (40%), squamous cell carcinoma (25–30%) and large cell carcinoma (5–10%).

The main methods of diagnosing lung cancer are computed tomography (CT) and positron emission computed tomography (PET/CT). [^18^F]F-FDG PET/CT provides more accurate N-staging in lung cancer than CT. Further, it is crucial to obtain sufficient material during a biopsy for histological verification of the diagnosis. In addition, liquid biopsy is now actively used to identify cancer biomarkers such as circulating tumor DNA, microRNA and circulating tumor cells.

Depending on the stage, surgery, radiation therapy, chemotherapy, immunotherapy, or molecular targeted therapy can be used to treat lung cancer [4]. Notably, immunotherapy and molecular targeted therapy began to be explicitly used to treat lung cancer in recent years.

Epithelial cell adhesion molecule (EpCAM) is currently being considered as a molecular target for immunotherapy of advanced cancer. An epithelial cell adhesion molecule is a type I transmembrane, calcium-independent glycoprotein, initially considered a cell adhesion molecule. However, current data show that it has only weak cell-adhesive properties [5,6]. EpCAM acts as a multifunctional transmembrane protein involved in regulating cell adhesion, proliferation, migration, stemness, and epithelial-to-mesenchymal transition (EMT) of normal and neoplastic epithelial cells. EpCAM has a prominent expression in multiple types of cancer and is considered a target for monoclonal antibody-mediated (mAb) cancer therapy, first and foremost in colorectal cancer [7,8]. Recently, a bispecific EpCAM/CD3 antibody has been developed as a trifunctional antibody Catumaxomab. The first results of clinical trials demonstrated efficacy, and the European Union approved Catumaxomab for treating patients with malignant ascites with EpCAM-positive carcinomas [9,10].

EpCAM overexpression is frequent in lung cancer [11]. Strong EpCAM immunostaining was detected in 85.7, 88.2 and 100% of adenocarcinoma, squamous cell carcinoma and small cell carcinoma of the lung, respectively [11]. It is also known today that noninvasive imaging of EpCAM expression has produced good results using nuclear medicine techniques in the case of renal cell carcinoma (RCC) in animals. In particular, engineered scaffold proteins (ESP) labeled with ^99m^Tc were used for radionuclide visualization of EpCAM expression [12]. The structure of ESPs determines their affinity for molecular targets. The developed proteins with ankyrin repeats (DARPins) are ESPs consisting of 4–6 blocks with a total molecular weight of 14–18 kDa. Previous studies have shown that DARPin Ec1 binds to EpCAM with a high affinity of 68 pM [13,14,15]. In addition, SPECT with engineering scaffold protein DARPin Ec1 labeled with technetium-99m has shown its effectiveness in imaging tumors with overexpression of EpCAM in preclinical studies and provided high contrast just a few hours after injection [16,17].

This first-in-human study aimed to evaluate the safety and distribution of [^99m^Tc]Tc(CO)_3_-(HE)_3_-Ec1 in patients with primary lung cancer. Three main objectives were set: first, to obtain basic information about the safety and tolerability of [^99m^Tc]Tc(CO)_3_-(HE)_3_-Ec1 after a single intravenous injection; second, to evaluate the distribution of [^99m^Tc]Tc(CO)_3_-(HE)_3_-Ec1 in normal tissues; and, third, to evaluate dosimetry of [^99m^Tc]Tc(CO)_3_-(HE)_3_-Ec1. A secondary but no less critical objective of this study was to study the possibility of imaging primary lung tumors and their metastases using [^99m^Tc]Tc(CO)_3_-(HE)_3_-Ec1 SPECT/CT.

## 2. Materials and Methods

### 2.1. Patients

This study was a prospective, open-label, non-randomized diagnostic study involving patients with untreated lung cancer (ClinicalTrials.gov ID: NCT05620472). The protocol of this clinical trial was approved by the Scientific Council of the Scientific Research Institute of Oncology and the Council on Medical Ethics of the Tomsk National Research Medical Center of the Russian Academy of Sciences (No. 18 dated 4 October 2022). All study participants signed written informed consent forms.

Twelve patients (Table 1) with verified lung cancer aged 36–72 years (nine males; three females) before surgical and chemotherapeutic treatment were enrolled in the study according to the following inclusion criteria: (1) clinical and radiological diagnosis of lung volumetric formation; (2) the size of the tumor measured by CT is more than 1 cm in the greatest diameter; (3) hematological, liver and renal function test results are within the normal limits; (4) negative pregnancy test for female patients of childbearing potential; (5) time after lung tumor biopsy is more than three weeks; and (6) capability to undergo the diagnostic radionuclide investigations planned as part of the study.

Patients were excluded from the study based on the following criteria: (1) active or remitted autoimmune disease; (2) active infection or history of severe infection; (3) known to be HIV-infected or have chronic active hepatitis B or C infection; (4) participation in other clinical trials; (5) claustrophobia.

Following the national standards of oncological care (Federal clinical guidelines for the diagnosis and treatment of lung cancer patients), all patients underwent a complete clinical examination including a CT of the chest with intravenous bolus contrast (Siemens Somatom Confidence, Munich, Germany). Lung tumor biopsy samples were taken in all patients included in the study; the diagnosis was confirmed using a morphological study. In all cases, immunohistochemistry (IHC) analysis of biopsy samples was performed for EpCAM detection.

### 2.2. Radiopharmaceutical

(HE)_3_-Ec1 was produced according to methods described earlier [13,17]. (HE)_3_-Ec1 was site-specifically labeled with technetium-99m by technetium tricarbonyl methodology using the protocol reported earlier [16,17]. After purification of ^99m^Tc-labeled (HE)_3_-Ec1 using NAP25 columns (Cytiva, Amersham, UK), the volume was adjusted to 10 mL using saline and sterilized by filtration. Radiochemical purity was analyzed by thin layer chromatography in PBS. The yield was 71 ± 8%, and the radiochemical purity was more than 97%. [^99m^Tc]Tc(CO)_3_-(HE)_3_-Ec1 was injected as an intravenous bolus. The injected protein dose was 3.0 mg of (HE)_3_-Ec1 for all patients. The average injected activity was 300.7 ± 103.2 MBq.

### 2.3. Evaluation of Safety and Tolerability

After a single intravenous injection of [^99m^Tc]Tc(CO)_3_-(HE)_3_-Ec1, all patients were under medical surveillance during the first 24 h. The general condition of the patients, body temperature, blood pressure, and heart function (electrocardiography) were evaluated. In addition, the general and biochemical parameters of blood and general parameters of urine before and 24 h, 48 h and seven days after injection of [^99m^Tc]Tc(CO)_3_-(HE)_3_-Ec1 were evaluated. Also, a medical examination of the patients was performed 48 h and seven days after injection of [^99m^Tc]Tc(CO)_3_-(HE)_3_-Ec1.

### 2.4. Imaging Protocol

All patients underwent SPECT/CT imaging. A Siemens Symbia Intevo Bold SPECT/CT hybrid scanner was used. A low-energy high-resolution (LEHR) collimator was used to obtain adequate image quality. Whole-body imaging in two planes was performed in planar mode (scan speed 12 cm/min, matrix 1024 × 256 pixels) and was performed in all patients at 2, 4, 6, and 24 h after injection. SPECT/CT lung scanning (SPECT: 60 planes, 20 s each, matrix 256 × 256 pixels; CT: 130 kV, effective 36 mAs) was performed at 2, 4, and 6 h after intravenous injection in all patients. For SPECT reconstruction, the standard xSPECT protocol (Siemens, Forchheim, Germany) based on the ordered subset conjugate gradient (OSCG) method (24 iterations, 2 subsets) was used. The 3D Gaussian FWHM 10 mm filter (Soft Tissue) was used. The proprietary software package syngo.via (Siemens, Forchheim, Germany) was used to process the obtained images.

### 2.5. Evaluation of Distribution and Dosimetry

To determine the accumulation level of [^99m^Tc]Tc(CO)_3_-(HE)_3_-Ec1 in the organs and throughout the body of patients, regions of interest (ROIs) were manually delineated in the anterior and posterior projections of the planar whole-body scans for each organ at each time point. The count rate was determined in each ROI. The count rate in a water-filled phantom containing ^99m^Tc-pertechnetate with known volumetric activity was measured to obtain a quantitative assessment. Chang’s correction was used for attenuation correction. The data from an ROI placed over the heart in the anterior and posterior projections were used to assess the activity in blood. The data were fitted to single exponential functions and residence times were calculated as the area under the fitted curves using Prism 9 (version 9.3.1, GraphPad Software, LLC, San Diego, CA, USA). Absorbed doses were calculated with OLINDA/EXM 1.1 software using adult female and male phantoms [18].

The maximal standard uptake value (SUV_max_) in tumor and nodal lesions with the highest [^99m^Tc]Tc(CO)_3_-(HE)_3_-Ec1 accumulation was calculated 2, 4, and 6 h after injection. The SUV_max_ in the contralateral region was determined to calculate the tumor-to-background SUV ratios.

### 2.6. Immunohistochemical Detection of EpCAM Expression

Immunohistochemical analysis for quantifying of EpCAM expression was performed using the Ventana BenchMark Ultra system (Roche Diagnostics, Tucson, AZ, USA). The protocol for the anti-EpCAM monoclonal antibody (clone Ber-EP4, Ventana Medical Systems, Inc., Tucson, AZ, USA) was meticulously followed during the staining of lung carcinoma biopsy samples. The samples were treated with Hematoxylin II and Bluing Reagent to achieve the necessary histological differentiation. Microscopic analysis was conducted using an Axio Imager M1 (Zeiss, Boston, MA, USA), where the expression of EpCAM was quantified in the tumor cells. The expression level was defined as a percentage of cells with strong membranous staining. The data were reported as the percentage of positively stained cells within ten high-power fields at 400× magnification.

### 2.7. Statistics

All statistical processing was carried out in Prism 10 (GraphPad Software, LLC, San Diego, CA, USA). The obtained values were described using mean ± standard deviation. To assess the significance of variations in uptakes across organs at different time points, we performed a one-way ANOVA analysis. Correlation analysis was conducted using Spearman’s criterion. In all tests, a significance level of *p* < 0.05 was considered significant.

## 3. Results

### 3.1. Safety and Tolerability

A single intravenous injection of [^99m^Tc]Tc(CO)_3_-(HE)_3_-Ec1 did not cause adverse events in the vital organs and systems of patients. The patients tolerated the injection well, and their somatic condition remained normal during the entire follow-up period. Patients did not actively present any complaints. According to instrumental and laboratory studies, the functional state of the organs and systems of patients did not differ before and after administration of [^99m^Tc]Tc(CO)_3_-(HE)_3_-Ec1. No changes were found in the blood or urine samples (Tables S1 and S2).

### 3.2. Evaluation of Distribution and Dosimetry

**Figure 1:**
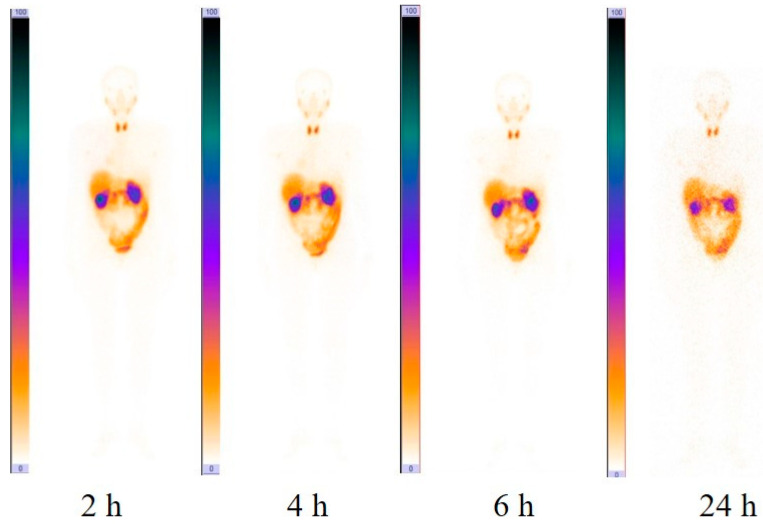
Anterior images of planar scintigraphy in patient 9 at 2, 4, 6 and 24 h after injection of [^99m^Tc]Tc(CO)_3_-(HE)_3_-Ec1. The upper setting of the scale window is 100% of the maximum counts.

**Figure 2:**
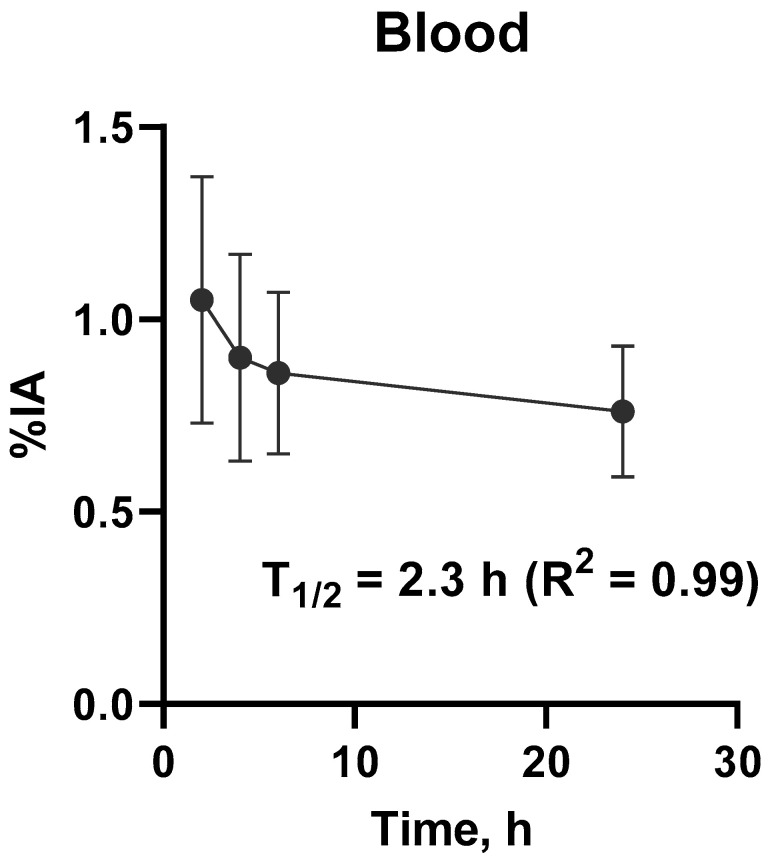
Kinetics of elimination of [^99m^Tc]Tc(CO)_3_-(HE)_3_-Ec1 from blood. Data are based on count rates in regions of interest placed over heart. %IA = percentage injected dose.

Liver and kidneys accumulated [^99m^Tc]Tc(CO)_3_-(HE)_3_-Ec1 most intensively (Table 2, Figure 1). There was also a noticeable accumulation of [^99m^Tc]Tc(CO)_3_-(HE)_3_-Ec1 in the lung, small and large intestines, pancreas and thyroid. However, the uptake in these organs was lower than 5% of injected activity per organ. The blood elimination half-life was 2.3 h (95% CI 1.3 to 16 h). The kinetics of [^99m^Tc]Tc(CO)_3_-(HE)_3_-Ec1 elimination from blood is shown in Figure 2.

The calculated values of absorbed doses in the main organs and tissues are shown in Table 3. The highest absorbed doses were in the kidneys, liver, pancreas, thyroid, gall bladder wall, and adrenals. The effective dose after a single intravenous injection of [^99m^Tc]Tc(CO)_3_-(HE)_3_-Ec1 was 0.011 ± 0.003 mSv/MBq. Thus, the effective dose for intravenous administration of the average activity in this study, 300 MBq, was 3.3 mSv.

### 3.3. [99^m^Tc]Tc(CO)_3_-(HE)_3_-Ec1 SPECT/CT Imaging of Primary Lung Tumors and Lymph Node Lesions

**Figure 3:**
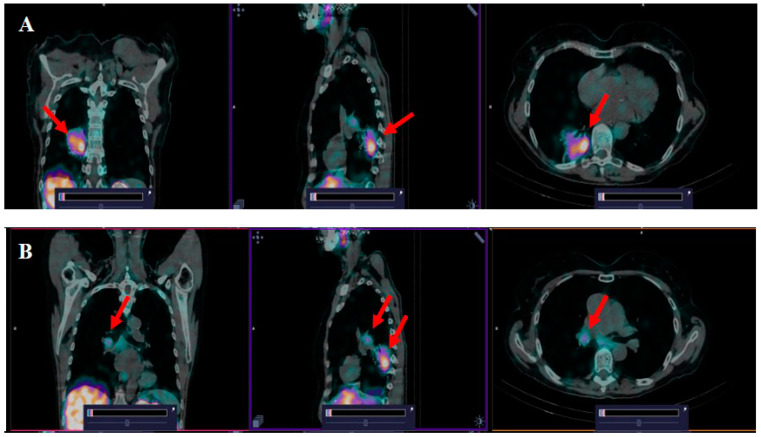
SPECT/CT images with [^99m^Tc]Tc(CO)_3_-(HE)_3_-Ec1 in patient 9 with cancer of the lower lobe of the right lung and regional lymph nodes metastases 2 h after injection. The upper setting of the SPECT scale window 5.66% of the maximum number. (**A**) The accumulation of [^99m^Tc]Tc(CO)_3_-(HE)_3_-Ec1 in the projection of the primary lung tumor (SUV_max_ = 6.8) (red arrows). (**B**) The accumulation of [^99m^Tc]Tc(CO)_3_-(HE)_3_-Ec1 in paratracheal lymph nodes affected by metastases (SUV_max_ = 2.47) (red arrows).

According to the SPECT/CT imaging, accumulation of [^99m^Tc]Tc(CO)_3_-(HE)_3_-Ec1 in the lung tumors was found in all patients included in the study. Intensive accumulation of [^99m^Tc]Tc(CO)_3_-(HE)_3_-Ec1 was also noted in regional metastases, which was compatible with the CT data concerning lymph node involvement (Figure 3). The calculated SUV_max_ in lung tumors (2, 4, 6 h) and SUV_max_ in regional metastases to lymph nodes (2 h) after injection of [^99m^Tc]Tc(CO)_3_-(HE)_3_-Ec1 are presented in Table 4.

The average level of [^99m^Tc]Tc(CO)_3_-(HE)_3_-Ec1 accumulation (SUV_max_) in lung tumors was 2.57 ± 1.52. At the same time, the accumulation of [^99m^Tc]Tc(CO)_3_-(HE)_3_-Ec1 in the intact lung tissue was appreciably lower. Therefore, the tumor-to-background ratios were high at all time points. This made it possible to clearly visualize the accumulation of [^99m^Tc]Tc(CO)_3_-(HE)_3_-Ec1 in lung tumors and metastases to regional lymph nodes, despite the relatively low values of SUV_max_. The difference in tumor/background ratios was practically not significant (*p* > 0.05, ANOVA test) at 2 h, 4 h and 6 h. This fact suggests that [^99m^Tc]Tc(CO)_3_-(HE)_3_-Ec1 SPECT/CT imaging of lung tumors with overexpression of EpCAM can be performed 2 h after intravenous injection. Moreover, imaging at a later time point does not improve imaging contrast.

**Figure 4:**
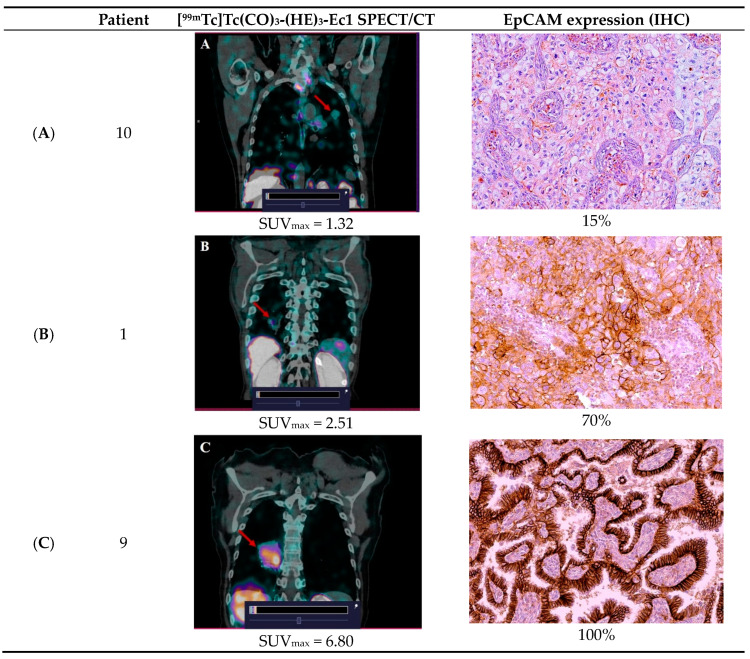
Comparison of SPECT/CT with [^99m^Tc]Tc(CO)_3_-(HE)_3_-Ec1 and EpCAM expression level in lung cancer patients. (**A**) SUV_max_ = 1.32 and EpCAM expression level—15%. (**B**) SUV_max_ = 2.51 and EpCAM expression level—70%. (**C**) SUV_max_ = 6.80 and EpCAM expression level—100%.

Comparison of SPECT/CT with [^99m^Tc]Tc(CO)_3_-(HE)_3_-Ec1 and IHC results revealed direct correlation between SUV_max_ values in primary tumor and the level of the EpCAM expression. For example, in the case of 100% EpCAM expression, SUV_max_ was 6.8; in 70% EpCAM expression, 2.51; and in 15%, 1.32 (Figure 4). This indicates that SPECT/CT imaging with [^99m^Tc]Tc(CO)_3_-(HE)_3_-Ec1 can possibly be used not only for visualization, but also for evaluation of the EpCAM expression level. Comparison of SPECT/CT with [^99m^Tc]Tc(CO)_3_-(HE)_3_-Ec1 and IHC results revealed that SUV_max_ value does not have a correlation with the proportion of EpCAM-positive cells (r = 0.82, *p* = 0.133), but this result may have limitations related to sample size.

## 4. Discussion

Today, the role of EpCAM in normal epithelial tissues in the development of epithelial tumors of various localizations has been studied in sufficient detail. Currently, EpCAM is still considered a specific prognostic cancer marker because it is involved in the processes of tumor progression and metastasis [19]. The high level of EpCAM expression in many malignant tumors prompted the active development of drugs for EpCAM-directed targeted therapy [20,21,22]. With this information, we can expect that more such therapeutic agents will be introduced into oncology practice in the near future. Therefore, we will need a reliable tool to effectively determine the indications for such therapy for various tumors with EpCAM overexpression. In recent years, radionuclide molecular imaging based on scaffold proteins has demonstrated its effectiveness as a relatively simple, non-invasive, sensitive and specific imaging method for the detection of expression of another therapeutic molecular target, HER2, in primary breast tumors and metastasis [23,24].

In the presented study, we demonstrated that [^99m^Tc]Tc(CO)_3_-(HE)_3_-Ec1, a novel probe for SPECT/CT imaging of tumors with EpCAM overexpression, is safe for human use and does not cause adverse events. It should be noted that the effective dose (0.011 ± 0.003 mSv/MBq) after injection of [^99m^Tc]Tc(CO)_3_-(HE)_3_-Ec1 allows it to be used in one patient several times a year. This enables, for example, the use of [^99m^Tc]Tc(CO)_3_-(HE)_3_-Ec1 SPECT/CT to evaluate the effectiveness of patient treatment.

Primary tumors were visualized in all patients included in the study, as exemplified in Figure 3. We found that the SUV_max_ values for primary tumors were variable and ranged from 1.1 to 6.8 (mean ± SD: 2.57 ± 1.52). In metastatic lymph nodes, the level of SUV_max_ was lower and amounted to 1.2 ± 0.5, but it still allowed for identification of the lesion. We cannot exclude that the lower uptake in metastases is due to the lower level of EpCAM expression in tumor cells. However, it might be that lower SUVs were due to incomplete partial volume effect correction in lymph node metastases, which were smaller than primary tumors. Notably, the tumor uptake level correlated with the expression of EpCAM in tumor biopsy samples (Figure 4). These data were confirmed by the determination of EpCAM in postoperative tumor samples. The number of patients enrolled in this study is too small for any profound conclusions. Our data are only an indication of the potential utility of [^99m^Tc]Tc(CO)_3_-(HE)_3_-Ec1-based imaging for evaluation of the target expression level. However, the data concerning the safety and tolerability of the tracer permit planning of a Phase II study to enable assessment of the accuracy of this methodology. Another limitation of this study is that only patients with non-small cell lung cancer were recruited. Small cell lung cancer is less noticeable. However, the pathology data show [11] that small cell lung cancer expresses high levels of EpCAM very frequently [25]. Accordingly, this should be evaluated in future studies. Furthermore, a comprehensive study will be required to study hypotheses that the tumor uptake values of [^99m^Tc]Tc(CO)_3_-(HE)_3_-Ec1 correlate with the response of lung cancer, as well as other malignancies to EpCAM-targeted therapies.

Earlier, ^111^In-labeled full-length IgG antibodies were investigated for imaging of lung cancer for staging purposes [26,27]. These studies included EpCAM-specific antibody MOC-31 [28] and demonstrated the capacity of such agents to visualize primary lung cancer tumors. However, the sensitivity of imaging of metastases was dependent on their size and location. Tumors smaller than 2 cm, as well as liver metastases, were frequently missed. This might be partially explained by the suboptimal imaging properties of indium-111, which requires the use of medium energy collimators. Such collimators reduce sensitivity and degrade spatial resolution. There is a more fundamental issue with the use of intact IgG. These bulky proteins clear from blood and extravasate slowly. The imaging might be performed not earlier than 2–3 days after injection [24]. Still, the contrast of imaging is limited, which is associated with low sensitivity. Imaging probes based on smaller Fab fragments were more successful in visualization. Thus, ^99m^Tc-labeled anti-EpCAM Fab fragment NR-LU-10 has been found to be a useful complement to CT-based staging [29,30]. However, NR-LU-10 is a murine antibody, and its use was associated with the development of human anti-mouse antibodies in 25% of patients, precluding its repetitive use. Despite these limitations, the cited studies demonstrated the potential of EpCAM-targeted radionuclide imaging in lung cancer and suggested that using smaller imaging probes is advantageous. DARPin Ec1 is approximately three-fold smaller than Fab. This offers the advantages of much more rapid localization in tumors and faster clearance from normal tissues. This study has demonstrated that a high contrast is reached only two hours after injection.

## 5. Conclusions

This first-in-human clinical study showed that injections of [^99m^Tc]Tc(CO)_3_-(HE)_3_-Ec1 are safe and tolerable. [^99m^Tc]Tc(CO)_3_-(HE)_3_-Ec1 SPECT/CT allows visualization of tumors with EpCAM overexpression and their metastases. Given the results obtained, [^99m^Tc]Tc(CO)_3_-(HE)_3_-Ec1 SPECT/CT should be further evaluated as a potential method to select patients for targeted anti-EpCAM therapy of tumors.

## Figures and Tables

**Table 1:** Characteristics of patients included in the clinical study.

Patient	Age (y)	Sex	Clinical Stage	Diagnosis (Histopathology)
1	71	Male	T3N0M0	PLC
2	58	Male	T3N0M0	IA
3	46	Male	T3N1M0	MDSCC
4	45	Male	T3N1M0	LDSCC
5	71	Male	T2N0M0	LDSCC
6	36	Male	T2N0M0	LDSCC
7	42	Male	T2N0M0	MDSCC
8	72	Male	T3N1M0	MDSCC
9	68	Female	T2N1M0	NMA
10	46	Male	T2N0M0	IA
11	47	Female	T1N2M0	MDSC
12	45	Female	T2N1M0	NMA

NMA—non-mucinous adenocarcinoma; IA—intestinal adenocarcinoma; PLC—pleomorphic lung carcinoma; MDSCC—moderately differentiated squamous cell carcinoma; LDSCC—low differentiated squamous cell carcinoma.

**Table 2:** Decay-corrected uptake of ^99m^Tc in the organs with the highest uptake based on planar imaging. The data are presented as average %ID ± SD per organ at different time points after injection of [^99m^Tc]Tc(CO)_3_-(HE)_3_-Ec1.

Time	Kidney	Liver	Lung
2 h	32.24 ± 9.63	10.38 ± 1.80	3.05 ± 0.86
4 h	30.68 ± 9.30	10.27 ± 1.83	2.82 ± 0.78
6 h	29.55 ± 8.83	10.02 ± 1.65	2.67 ± 0.78
24 h	22.1 ± 5.58	9.12 ± 1.55	2.51 ± 0.80

**Table 3:** Absorbed doses (mGy/MBq) after injection of [^99m^Tc]Tc(CO)_3_-(HE)_3_-Ec1.

Site	Absorbed Dose
Adrenals	0.017 ± 0.004
Brain	0.001 ± 0.000
Breasts	0.002 ± 0.000
Gallbladder Wall	0.010 ± 0.002
LLI Wall	0.006 ± 0.002
Small Intestine	0.007 ± 0.002
Stomach Wall	0.005 ± 0.001
ULI Wall	0.006 ± 0.002
Heart Wall	0.005 ± 0.001
Kidneys	0.113 ± 0.044
Liver	0.013 ± 0.002
Lungs	0.005 ± 0.001
Muscle	0.003 ± 0.001
Ovaries	0.004 ± 0.001
Pancreas	0.010 ± 0.002
Red Marrow	0.005 ± 0.004
Osteogenic Cells	0.007 ± 0.001
Skin	0.003 ± 0.003
Spleen	0.008 ± 0.004
Testes	0.006 ± 0.003
Thymus	0.004 ± 0.001
Thyroid	0.056 ± 0.012
Urinary Bladder Wall	0.009 ± 0.008
Prostate	0.005 ± 0.002
Total body	0.004 ± 0.001
Effective Dose Equivalent (mSv/MBq)	0.015 ± 0.004
Effective Dose (mSv/MBq)	0.011 ± 0.003

**Table 4:** SUV_max_ (at 2 h), SUV tumor-to-background (at 2, 4 and 6 h) and SUV_max_ lymph nodes (at 2 h) after the injections of [^99m^Tc]Tc(CO)_3_-(HE)_3_-Ec1.

Patients	SUV_max Tumor_	SUV_max_ _Lymph Node_	SUV_Tumor/Background 2 h_	SUV_Tumor/Background 4 h_	SUV_Tumor/Background 6 h_
1.	2.51	-	7.17	6.4	7
2.	2.8	-	23.3	13.2	13.4
3.	2.29	0.76	10.9	10.3	14.4
4.	1.42	0.79	8.35	8.3	6.5
5.	1.1	-	6.88	7.2	10.4
6.	3.23	-	7.17	5	12.3
7.	2.83	-	14.9	15.4	16.5
8.	1.16	0.94	9.7	10.5	9.4
9.	6.8	2.47	27.2	22	26.3
10.	1.32	-	12	7.6	2.3
11.	2.73	1.75	15.2	5.7	6.4
12.	2.66	1.82	20.5	13.3	13.2
Mean ± SD	2.57 ± 1.52	1.2 ± 0.5	13.6 ± 6.81	10.4 ± 4.91	11.5 ± 6.2

## Data Availability

The data generated during the current study are available from the corresponding author upon reasonable request.

## Supplementary Materials

The following supporting information can be downloaded at: https://www.mdpi.com/article/10.3390/cancers16162815/s1, Table S1: Parameters of blood biochemistry in patients before intravenous administration of a radiopharmaceutical and after 24, 48 h and 7 days post administration; Table S2: Complete blood count in patients before intravenous administration of the radiopharmaceutical and 24, 48 h and 7 days after administration.

## References

[1] Jones G.S., Baldwin D.R. (2018). Recent advances in the management of lung cancer. Clin. Med..

[2] Bade B.C., Dela Cruz C.S. (2020). Lung Cancer 2020: Epidemiology, Etiology, and Prevention. Clin. Chest Med..

[3] Alduais Y., Zhang H., Fan F., Chen J., Chen B. (2023). Non-small cell lung cancer (NSCLC): A review of risk factors, diagnosis, and treatment. Medicine.

[4] Pignon J.P., Tribodet H., Scagliotti G.V., Douillard J.Y., Shepherd F.A., Stephens R.J., Dunant A., Torri V., Rosell R., Seymour L. (2008). Lung adjuvant cisplatin evaluation: A pooled analysis by the LACE Collaborative Group. J. Clin. Oncol. Off. J. Am. Soc. Clin. Oncol..

[5] Koprowski H., Steplewski Z., Mitchell K., Herlyn M., Herlyn D., Fuhrer P. (1979). Colorectal carcinoma antigens detected by hybridoma antibodies. Somat. Cell Genet..

[6] Litvinov S.V., Balzar M., Winter M.J., Bakker H.A., Briaire-de Bruijn I.H., Prins F., Fleuren G.J., Warnaar S.O. (1997). Epithelial cell adhesion molecule (Ep-CAM) modulates cell-cell interactions mediated by classic cadherins. J. Cell Biol..

[7] Eyvazi S., Farajnia S., Dastmalchi S., Kanipour F., Zarredar H., Bandehpour M. (2018). Antibody Based EpCAM Targeted Therapy of Cancer, Review and Update. Curr. Cancer Drug Targets.

[8] Gires O., Pan M., Schinke H., Canis M., Baeuerle P.A. (2020). Expression and function of epithelial cell adhesion molecule EpCAM: Where are we after 40 years?. Cancer Metastasis Rev..

[9] Knödler M., Körfer J., Kunzmann V., Trojan J., Daum S., Schenk M., Kullmann F., Schroll S., Behringer D., Stahl M. (2018). Randomised phase II trial to investigate catumaxomab (anti-EpCAM × anti-CD3) for treatment of peritoneal carcinomatosis in patients with gastric cancer. Br. J. Cancer.

[10] Ruf P., Kluge M., Jäger M., Burges A., Volovat C., Heiss M.M., Hess J., Wimberger P., Brandt B., Lindhofer H. (2010). Pharmacokinetics, immunogenicity and bioactivity of the therapeutic antibody catumaxomab intraperitoneally administered to cancer patients. Br. J. Clin. Pharmacol..

[11] Menz A., Lony N., Lennartz M., Dwertmann Rico S., Schlichter R., Kind S., Reiswich V., Viehweger F., Dum D., Luebke A.M. (2024). Epithelial Cell Adhesion Molecule (EpCAM) Expression in Human Tumors: A Comparison with Pan-Cytokeratin and TROP2 in 14,832 Tumors. Diagnostics.

[12] Tolmachev V., Bodenko V., Orlova A., Schulga A., Deyev S.M., Vorobyeva A. (2023). Visualization of epithelial cell adhesion molecule-expressing renal cell carcinoma xenografts using designed ankyrin repeat protein Ec1 labelled with ^99m^Tc and ^125^I. Oncol. Lett..

[13] Stefan N., Martin-Killias P., Wyss-Stoeckle S., Honegger A., Zangemeister-Wittke U., Plückthun A. (2011). DARPins recognizing the tumor-associated antigen EpCAM selected by phage and ribosome display and engineered for multivalency. J. Mol. Biol..

[14] Tolmachev V.M., Chernov V.I., Deyev S.M. (2022). Targeted nuclear medicine. Seek and destroy. Russ. Chem. Rev..

[15] Plückthun A. (2015). Designed ankyrin repeat proteins (DARPins): Binding proteins for research, diagnostics, and therapy. Annu. Rev. Pharmacol. Toxicol..

[16] Vorobyeva A., Bezverkhniaia E., Konovalova E., Schulga A., Garousi J., Vorontsova O., Abouzayed A., Orlova A., Deyev S., Tolmachev V. (2020). Radionuclide Molecular Imaging of EpCAM Expression in Triple-Negative Breast Cancer Using the Scaffold Protein DARPin Ec1. Molecules.

[17] Deyev S.M., Vorobyeva A., Schulga A., Abouzayed A., Günther T., Garousi J., Konovalova E., Ding H., Gräslund T., Orlova A. (2020). Effect of a radiolabel biochemical nature on tumor-targeting properties of EpCAM-binding engineered scaffold protein DARPin Ec1. Int. J. Biol. Macromol..

[18] Chernov V., Dudnikova E., Zelchan R., Medvedeva A., Rybina A., Bragina O., Goldberg V., Muravleva A., Sörensen J., Tolmachev V. (2022). Phase I Clinical Trial Using [^99m^Tc]Tc-1-thio-D-glucose for Diagnosis of Lymphoma Patients. Pharmaceutics.

[19] Li G., Suzuki H., Asano T., Tanaka T., Suzuki H., Kaneko M.K., Kato Y. (2022). Development of a Novel Anti-EpCAM Monoclonal Antibody for Various Applications. Antibodies.

[20] Li G., Suzuki H., Ohishi T., Asano T., Tanaka T., Yanaka M., Nakamura T., Yoshikawa T., Kawada M., Kaneko M.K. (2023). Antitumor activities of a defucosylated anti-EpCAM monoclonal antibody in colorectal carcinoma xenograft models. Int. J. Mol. Med..

[21] Satofuka H., Wang Y., Yamazaki K., Hamamichi S., Fukuhara T., Rafique A., Osako N., Kanazawa I., Endo T., Miyake N. (2023). Characterization of human anti-EpCAM antibodies for developing an antibody-drug conjugate. Sci. Rep..

[22] Xu T., Karschnia P., Cadilha B.L., Dede S., Lorenz M., Seewaldt N., Nikolaishvili E., Müller K., Blobner J., Teske N. (2023). In vivo dynamics and anti-tumor effects of EpCAM-directed CAR T-cells against brain metastases from lung cancer. Oncoimmunology.

[23] Bragina O., Chernov V., Larkina M., Rybina A., Zelchan R., Garbukov E., Oroujeni M., Loftenius A., Orlova A., Sörensen J. (2023). Phase I clinical evaluation of ^99m^Tc-labeled Affibody molecule for imaging HER2 expression in breast cancer. Theranostics.

[24] Tolmachev V., Orlova A., Andersson K. (2014). Methods for radiolabelling of monoclonal antibodies. Methods Mol. Biol..

[25] Zhu W.F., Li J., Yu L.C., Wu Y., Tang X.P., Hu Y.M., Chen Y.C. (2014). Prognostic value of EpCAM/MUC1 mRNA-positive cells in non-small cell lung cancer patients. Tumour Biol..

[26] Breitz H.B., Sullivan K., Nelp W.B. (1993). Imaging lung cancer with radiolabeled antibodies. Semin. Nucl. Med..

[27] Kalofonos H.P., Sivolapenko G.B., Courtenay-Luck N.S., Snook D.E., Hooker G.R., Winter R., McKenzie C.G., Taylor-Papadimitriou J.J., Lavender P.J., Epenetos A.A. (1988). Antibody guided targeting of non-small cell lung cancer using 111In-labeled HMFG1 F(ab’)2 fragments. Cancer Res..

[28] Kosterink J.G., de Jonge M.W., Smit E.F., Piers D.A., Kengen R.A., Postmus P.E., Shochat D., Groen H.J., The H.T., de Leij L. (1995). Pharmacokinetics and scintigraphy of indium-111-DTPA-MOC-31 in small-cell lung carcinoma. J. Nucl. Med. Off. Publ. Soc. Nucl. Med..

[29] Friedman S., Sullivan K., Salk D., Nelp W.B., Griep R.J., Johnson D.H., Blend M.J., Aye R., Suppers V., Abrams P.G. (1990). Staging non-small cell carcinoma of the lung using technetium-99m-labeled monoclonal antibodies. Hematol./Oncol. Clin. N. Am..

[30] Vansant J.P., Johnson D.H., O’Donnell D.M., Stewart J.R., Sonin A.H., McCook B.M., Powers T.A., Salk D.J., Frist W.H., Sandler M.P. (1992). Staging lung carcinoma with a Tc-99m labeled monoclonal antibody. Clin. Nucl. Med..

