# Supplementary material for: Phase I Clinical Evaluation of Designed Ankyrin Repeat Protein [99mTc]Tc(CO)3-(HE)3-Ec1 for Visualization of EpCAM-Expressing Lung Cancer
Source: Cancers (Basel). 2024 Aug 10;16(16):2815. doi: 10.3390/cancers16162815 (PMC11353007; doi:10.3390/cancers16162815)
Supplement: Supplementary file 1 [file cancers-16-02815-s001.zip › cancers-3116405-supplementary.pdf]

Supplementary Materials: Phase I Clinical Evaluation of Designed Ankyrin Repeat Protein [^{99m}Tc]Tc(CO)$_3$-(HE)$_3$-Ec1 for Visualization of EpCAM-Expressing Lung Cancer

Table S1. Parameters of blood biochemistry in patients before intravenous administration of a radiopharmaceutical and after 24, 48 hours and 7 days post administration.

Blood index	Before intravenous administration Me (Q1–Q3)	After a single intravenous administration		
		24 h Me (Q1–Q3)	48 h Me (Q1–Q3)	7 days Me (Q1–Q3)
Alanine aminotransferase, U/L	14.0 (12–15)	13.0 (11–16) p = 0.25	13.0 (12–15) p = 0.26	12.0 (11–16) p = 0.25
Aspartate aminotransferase, U/L	14.0 (12–16)	14.0 (12–17) p = 0.31	15.0 (12–16) p = 0.32	15.0 (13–16) p = 0.29
Alkaline phosphatase, U/L	161.0 (145–200)	161.0 (143–200) p = 0.21	162.0 (151–205) p = 0.25	161.0 (151–206) p = 0.24
Glucose, mmol/L	4.6 (4.2–5.6)	4.5 (4.2–5.4) p = 0.22	4.6 (4.1–5.4) p = 0.22	4.6 (4.1–5.4) p = 0.25
Creatinine, µmol/L	85.0 (66–89)	87.0 (68–92) p = 0.24	85.0 (66–90) p = 0.28	85.0 (68–93) p = 0.31
Protein, g/L	73.0 (64–75)	73.5 (66–74) p = 0.31	73.0 (65–75) p = 0.24	74.0 (67–77) p = 0.29
Bilirubin, µmol/L	9.8 (6.0–12.5)	9.8 (6.5–12.6) p = 0.22	9.8 (6.2–12.5) p = 0.33	9.8 (5.6–12.9) p = 0.36

Table S2. Complete blood count in patients before intravenous administration of the radiopharmaceutical and 24, 48 hours and 7 days after administration.

Blood index	Before intravenous administration [$Q_1 – Q_3$]	After a single intravenous administration		
		**24 h Me [$Q_1 – Q_3$]**	**48 h Me [$Q_1 – Q_3$]**	**7 days Me [$Q_1 – Q_3$]**
Hemoglobin, g/l	120,6 [117–129]	120,2 [119–130] p = 0,31	122,0 [118–127] p = 0,19	123,0 [119–131] p = 0,22
Red blood cells, 10^{12}/l	4,1 [3,9-4,5]	4,1 [3,8–4,2] p = 1,00	4,1 [3,8–4,2] p = 0,8	4,3 [3,9–4,3] p = 1,00
Platelets, 10^9/l	244,0 [195-247]	239,0 [195–246] p= 0,4	239,0 [195–246] p= 0,45	242,6 [198–246] p= 0,4
Erythrocyte sedimentation rat, mm/h	3,2[1-6]	3,1 [1–6] p= 0,1	3,2 [1–5] p= 0,22	3,1 [1–5] p= 0,23
White blood cells, 10^{12}/l	5,9 [4,2-6,3]	5,8 [4,5–6,0] p=0,8	5,7 [4,4–6,0] p=1,00	5,8 [5,0–6,1] p=1,00
Eosinophils, %	2,0 [1-2]	2,0 [1–3] p= 0,16	2,0 [1–4] p= 0,16	2,0 [1–3] p= 0,14
Basophils, %	0,0 [0-1]	0,0 [0–1] p = 0,07	0,0 [0–1] p = 0,07	0,0 [0–1] p = 0,07
Monocytes, %	4,0 [3-5]	4,0 [3–5] p = 0,06	4,0 [3–5] p = 0,06	3,5 [3–5] p = 0,2
Lymphocytes, %	26,0 [22-33]	24,0 [21–31] p = 0,25	25,0 [22–32] p = 0,18	26,0 [23–32] p = 0,19
